# *FLAP*: a framework for linking free-text addresses to the Ordnance Survey Unique Property Reference Number database

**DOI:** 10.3389/fdgth.2023.1186208

**Published:** 2023-11-28

**Authors:** Huayu Zhang, Arlene Casey, Imane Guellil, Víctor Suárez-Paniagua, Clare MacRae, Charis Marwick, Honghan Wu, Bruce Guthrie, Beatrice Alex

**Affiliations:** ^1^ Advanced Care Research Centre, The Usher Institute, College of Medicine and Veterinary Medicine, University of Edinburgh, Edinburgh, United Kingdom; ^2^ Deanery of Molecular, Genetic and Population Health Sciences, Usher Institute, College of Medicine and Veterinary Medicine, University of Edinburgh, Edinburgh, United Kingdom; ^3^ Population Health and Genomics, School of Medicine, University of Dundee, Dundee, United Kingdom; ^4^ Institute of Health Informatics, University College London, London, United Kingdom; ^5^ School of Literatures, Languages and Cultures, University of Edinburgh, Edinburgh, United Kingdom; ^6^ Edinburgh Futures Institute, University of Edinburgh, Edinburgh, United Kingdom

**Keywords:** free-text address, Unique Property Reference Number, UPRN, record linkage, machine learning

## Abstract

**Introduction:**

Linking free-text addresses to unique identifiers in a structural address database [the Ordnance Survey unique property reference number (UPRN) in the United Kingdom (UK)] is a necessary step for downstream geospatial analysis in many digital health systems, e.g., for identification of care home residents, understanding housing transitions in later life, and informing decision making on geographical health and social care resource distribution. However, there is a lack of open-source tools for this task with performance validated in a test data set.

**Methods:**

In this article, we propose a generalisable solution (A **F**ramework for **L**inking free-text **A**ddresses to Ordnance Survey U**P**RN database, *FLAP*) based on a machine learning–based matching classifier coupled with a fuzzy aligning algorithm for feature generation with better performance than existing tools. The framework is implemented in Python as an Open Source tool (available at *Link*). We tested the framework in a real-world scenario of linking individual’s (n=771,588) addresses recorded as free text in the Community Health Index (CHI) of National Health Service (NHS) Tayside and NHS Fife to the Unique Property Reference Number database (UPRN DB).

**Results:**

We achieved an adjusted matching accuracy of 0.992 in a test data set randomly sampled (n=3,876) from NHS Tayside and NHS Fife CHI addresses. *FLAP* showed robustness against input variations including typographical errors, alternative formats, and partially incorrect information. It has also improved usability compared to existing solutions allowing the use of a customised threshold of matching confidence and selection of top n candidate records. The use of machine learning also provides better adaptability of the tool to new data and enables continuous improvement.

**Discussion:**

In conclusion, we have developed a framework, *FLAP*, for linking free-text UK addresses to the UPRN DB with good performance and usability in a real-world task.

## 1. Introduction

Linkage of free-text addresses to a structural address database is necessary for downstream tasks relying on the geospatial attributes of individuals and other entities. In the health and social care context, such tasks include using service-recorded addresses to identify care home residents or homeless people, understand housing transitions in later life, and inform geographical health and social care resource distribution. The COVID-19 pandemic highlighted the value of this linkage, where understanding of geographical patterns of infection and impact on care homes was supported by address-matching to an Ordnance Survey reference database, an example being the analysis of geographical clustering of cases (1–4).

**Figure 1:**
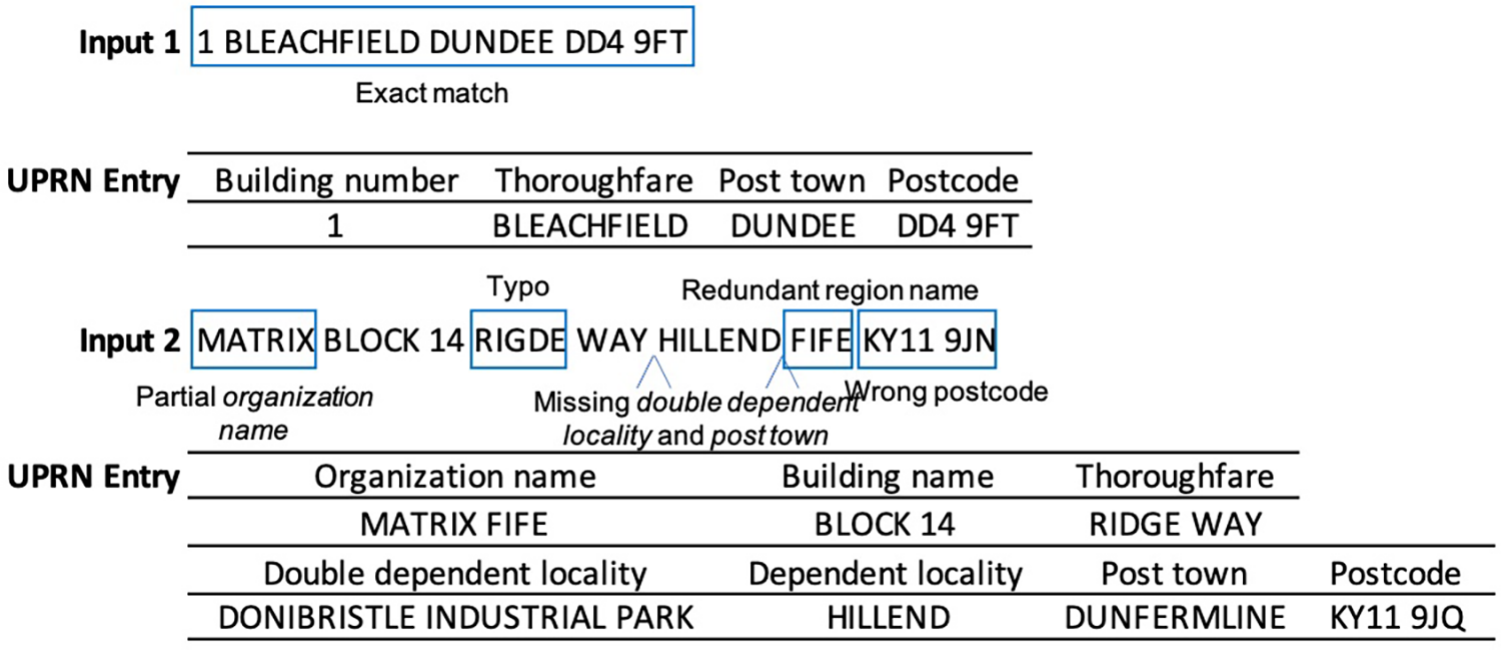
Examples of input address matched to records in the Ordnance Survey Unique Property Reference Number (UPRN) database (DB). The UPRN DB contains fields with textual address information (empty UPRN fields are not shown here). Input 1 matches exactly to record with a UPRN entry. Input 2 has several deviations from the standard address recorded in the UPRN database: text corresponding to *ORGANISATION NAME* partially missing, typographical errors in text corresponding to *THOROUGHFARE*, missing texts corresponding to *DOUBLE DEPENDENT LOCALITY* and *POST TOWN*, redundant information of region which is not recorded in the UPRN DB, and wrong *POSTCODE*.

The task is a record linkage problem, defined as the problem of finding records referring to the same entities across different data sources. Similar to traditional record linkage tasks, the solution needs to deal with situations when input data are incomplete or not in the database. Linking free-text addresses to database records has extra challenges compared to traditional record linkage tasks. Since NHS address input is semi-structured, there is no prior knowledge of the position of matching information to fields of structural records. There are also many possible variations in the input, such as typographical errors, abbreviations, and alternative forms (e.g., *5/1 Brunswick Road* could also be written as *Flat 1, 5 Brunswick Rd* or *Flat 1, 5 Brunswick Road*). Finally, the free-text addresses are created by GP practices, and local knowledge may result in addresses being recorded in a non-standard but locally understandable way (e.g., “Ron Sealey Volvo Specialist, Cowdenbeath” corresponds to “17 Wilson Street, Cowdenbeath KY4 9DQ” in the standard database) (Figure 1).

Different types of methods have been studied for record linkage including deterministic record linkage, probabilistic record linkage, and machine learning algorithms. Deterministic record linkage (also called rule-based record linkage) is the simplest type of record linkage. Deterministic record linkage aims to generate a rule or sets of rules, which determine if two records refer to the same entity (5). Probabilistic record linkage (also called fuzzy matching) calculates the probability that two given records refer to the same entity based on potential identifiers and weights of the identifiers (6). In recent years, machine learning has been used in record linkage (7). Machine learning algorithms are able to learn more complex patterns in potential identifiers (or features in machine learning terminology) and output probabilistic statements on whether a pair of records links. While training machine learning algorithm requires the preparation of labelled data similar to the development of other methods, the training process of machine learning algorithms require minimal human intervention, therefore reducing time for developing and adapting a method to new data.

In the UK, a standard address database (referred to as the Unique Property Reference Number database or UPRN DB) is maintained by Ordnance Survey. The textual addresses are presented with 11 fields, such as *ORGANISATION NAME*, *THOROUGHFARE*, and *POSTCODE*. Each record in the UPRN DB has a unique identifier, namely, the UPRN. Currently, there are several tools for free-text address to UPRN matching task. The *CHI-UPRN Residential Linkage (CURL)* tool is a deterministic record linkage method developed by Clark et al. for the same task (8). CURL was developed using address fields (three address lines plus postcode) extracted from the Public Health Scotland (PHS) Community Health Index (CHI) monthly download (dated 03 August 2020). CURL consists of minimal preprocessing for formatting and abbreviation expansion. The addresses were linked to UPRN identifiers using a set of rules (exact rules were not provided with the report). The tool was able to match more than 89% of the records in the development data, but its performance was not evaluated using labelled data. The true performance has therefore not been determined. The *ASSIGN* tool is a deterministic record linkage method developed by Harper et al (9). *ASSIGN* was developed using local authority–sourced addresses in Wales and from the London Borough of Tower Hamlets in east London. It implemented a preprocessing step, which is comprised of formatting and parsing. The parsed fields, namely, *Post code*, *Street*, *Number*, *Building*, and *Flat*, are matched to records in the UPRN database. A set of 29 rules with descending fitness were created to rank possible matches between input address and candidate records. The tool achieved 0.995 and 0.996 match rates with a sensitivity of 0.999 and 0.998 and a positive predictive value of 0.996 and 0.998 in the Welsh and London derivation datasets, respectively. A 0.986 match rate for a test population of seven northeast London Clinical Commissioning Groups (CCGs, the test data set) was achieved, but the accuracy of matching in the test data set was not assessed. Proprietary tools are available for address-UPRN matching tasks, such as Esri UK (10) and Loqate GBG (11), but they are not publicly evaluated, to the best of our knowledge. In summary, there is a lack of open-source tools for this task with performance validated in a test data set. Ideally, the tool should be adaptable to new data. Usability of the existing methods can also be improved by introducing a confidence score, so that the users could choose a threshold that better meet the requirement of precision and recall in their downstream tasks.

The aim of this study is to develop and validate a generalisable framework for linking free-text addresses to a structural address database. In this paper, We described the method used for the framework *FLAP* (A **F**ramework for **L**inking free-text **A**ddresses to Ordnance Survey U**P**RN database). We reported the performance of the framework in a real-world problem of linking Community Health Index (CHI) addresses in National Health Service (NHS) Tayside and NHS Fife to the UPRN DB.

## 2. Material and methods

### 2.1. Definition of the task

The address-UPRN linking task is defined as follows: Given an address input/UPRN DB record pair (s,d), determine whether the pair is a matching record. The address input s is a sequence of characters and the UPRN DB record d is a dictionary with each element being a pair of UPRN DB field name and a sequence of characters (Figure 1).

### 2.2. Database augmentation

Some input variations of addresses cannot be resolved with sequence alignment, since alternative forms of the same address do not necessarily have sequential textual similarity (e.g., *“5/1”* could be written as *“Flat 1, 5”*).

To meet this challenge, we can generate alternative forms of addresses from standard ones using a sequence transformation process. Transforming addresses for mapping their alternative forms to the standard form in the UPRN DB can be done either by trying to normalise input addresses or by deriving alternative forms of addresses from the standard address database. We implemented the latter approach for the following reasons: (1) There is no ambiguity of synonym use in standard addresses (*“ST.”* stands for *“Saint”* in the UPRN DB, while in input address *“ST”* or *“ST.”* can refer to *“Saint”* but *“ST”* can alternatively and more commonly mean *“STREET”*). (2) Standard addresses offer structural fields, while input addresses are semi-structured. Applying transformations to input addresses, therefore, requires field parsing, which adds another layer of complexity. Importantly, the database augmentation process can be independently developed and used. Please refer to Supplementary Tables S1 and S2 for the list of transformations used for database augmentation.

The database augmentation process can also be used to include local knowledge of addresses. Local knowledge is the knowledge of two textually unrelated addresses referring to the same address. For example, the name of a building in a rural area is normally enough to be linked to a unique UPRN ID, but this name is not necessarily included in the UPRN DB (e.g., *“Roselea House Cowdenbeath”* can be matched to *“175 STENHOUSE STREET COWDENBEATH”* in the UPRN database).

Consistency checking was carried out to ensure that no duplicated records were created in the database augmentation process.

### 2.3. Input preprocessing

There are two steps of preprocessing used in our framework: elimination of irregular characters and knowledge-based completion or deletion. Elimination of irregular characters removes characters that are not common in address strings (e.g., special characters other than “-” or “/” and multiple spaces). Knowledge-based completion aims to complete the input address or remove redundant information based on knowledge inferred from the UPRN DB. For example, if an address contains the subsumption of a region (e.g., a *DEPENDENT LOCALITY* within a *POST TOWN*) but not the region itself, the region was added to the input address (e.g., *“272 HIGH STREET, METHIL, KY8 3EQ”* to *“272 HIGH STREET, METHIL, LEVEN, KY8 3EQ”*). Elements that are not used by UPRN DB were removed (e.g., *“RIVERSIDE ROAD, LEVEL, FIFE, KY8 4LT”* to *“RIVERSIDE ROAD, LEVEL, KY8 4LT”*).

### 2.4. Alignment of UPRN DB fields to an input address

Since the input addresses are in the form of free text, alignment is required to select the best possible candidate character sequence (and its position) in the input sequences by considering the input variations (e.g., typographical errors or word concatenation). Features of the alignment quality such as the positions of match, insertion, and mismatch were kept.

While there are multiple ways to implement fuzzy alignments of sequences, we adopted an approach combining the Needleman–Wunsch sequence alignment algorithm (NW-alignment) (12) with Damerau–Levenshtein distance (DL-distance) (13) for easy implementation in safe havens (no dependency on external libraries). Sequences are aligned twice using a token-based and a string-based approach. The better of the two alignments is chosen.

For the token-based approach, the sequences were first tokenised using the regular expression “not word” (“\W”), and then aligned using NW-alignment. A threshold for maximum DL-distance was set to 0.2. The threshold corresponds to less than one error in five letters. The threshold was chosen empirically due to the observation that typographical errors typically occurred in words longer than five letters. If the DL-distance of two tokens was below the threshold, they were considered to be the same token, which allowed fuzziness in the token-based sequence alignment. The token-based alignment approach can be potentially extended to consider semantic similarities of tokens using word embedding vectors.

For the string-based approach, the sequences were aligned in the original form using NW-alignment. A post-processing step is added to limit the non-informative alignment of characters based on the alignment percentage in the token spans. For each token span, the string-based alignment is discarded if the percentage of characters aligned in the span is less than 80%, which is equivalent to the DL-distance threshold of 0.2 in token alignment. The string-based approach is essential in situations like word concatenation (e.g. “GREENBANK” cannot be aligned to “GREEN BANK” with the token-based method but can be aligned with the string-based one).

The following metrics were calculated during the alignment process for each UPRN field that is not blank: percentage aligned in the input address, percentage aligned in the UPRN field, the harmonic mean of the two percentages M, character frequency cosine similarity F, and the number of insertions I. A summary score was calculated as S=M−0.01×I+0.1×F.

The alignment result with the higher summary score was chosen (from the two approaches).

### 2.5. Conversion of UPRN DB to Tree DB and searching the Tree DB for candidate UPRN records

Information that is better formatted and more structural in the input free text can be used to limit the search space to records in the UPRN DB corresponding to a small area, in which one can exhaustively match all candidates in a reasonable runtime.

We generalised the tasks of limiting the search space to a local area and exhaustive search in this local area to depth-first search (DFS) and breadth-first search (BFS) in a tree-structured database, respectively. From the prospective of record linkage, our strategy was equivalent to blocking using filters.

The conversion of UPRN DB to Tree DB follows the rules: (1) Each layer of the tree corresponds to a field (columns) in the UPRN DB and the order of the layers is set in advance. (2) The UPRN DB is attached to the root of the tree. (3) For each node of the current layer, the UPRN DB is split by unique values of the corresponding field. For each unique value and the corresponding slice of the UPRN DB, a child node is created with the name of the unique value and the slice of the UPRN DB is attached. (4) The UPRN DB is deleted from the parent node. (5) Steps (3) and (4) are iterated until the last layer, at which point, each leaf node corresponds to one UPRN DB record (Supplementary Figure S1).

For DFS, the child node with the best summary score (see Section 2.4) from the alignment of the node name with the input address string was chosen. DFS was done for one step using parsed postcode or for two steps using parsed post town and thoroughfare. The chosen best child node from DFS was then passed to BFS. For BFS, the names of all children nodes were aligned to the input address string and move on to the next layer of the tree until the leaf nodes are reached. For each alignment of the node name with the input address string, aligned positions in the input address string were redacted and passed on to the children nodes. At each leaf node, alignment metrics of the input string to all UPRN fields were collected by tracing back to the root of the Tree DB. These metrics served as input to the matching classifier (see Section 2.6).

### 2.6. The matching classifier

The matching classifier is a binary classifier predicting whether an input address is an equivalent entity of a UPRN DB record. We chose the *Random Forest Classifier* for this task for three reasons. First, there are many zero-valued features because a UPRN DB record normally contains some but not all of the UPRN fields. The ensemble setup of the Random Forest Classifier increases the chance that some classification trees utilise the important features during the random feature selection process. Second, for matching records, all matching metrics are expected to be higher. Therefore, there is high co-linearity among the features, which is not a concern for the *Random Forest Classifier*. Finally, the hardware requirement of the classifier is low.

The alignment process in Section 2.4 generates 52 features as the input to the matching classifier. A summary of all features can be found in Table 1. For supervised training of the matching classifier, we assembled labelled data of true matches and false matches as the training data set. Matching addresses from manual annotation (see Section 2.10) served as true matches. False matches were sampled by generating pairs of the input address and UPRN records in the same postcode excluding the correct UPRN match.

**Table 1:** The summary of features for training the matching classifier.

Description	Source	*N* feature(s)
% aligned in the input address	Each UPRN field	11
% aligned in the UPRN field	Each UPRN field	11
*N* of insertions in alignments	Each UPRN field	11
Character frequency cosine similarity	Each UPRN field	11
Mean character frequency cosine similarity	Overall	1
Digits in alignment residual of input address	Overall	1
Single character in alignment residual of input address	Overall	1
Digits in alignment residual of UPRN fields	Overall	1
Single character in alignment residual of UPRN fields	Overall	1
Sum of % aligned in the input address	Overall	1
Sum of % aligned in the UPRN fields	Overall	1
Sum of harmonic means	Overall	1
	Total	52

The probability output of the *Random Forest Classifier* was used as the summary score for the matching between an address input and a UPRN DB record.

### 2.7. The matching work flow

**Figure 2:**
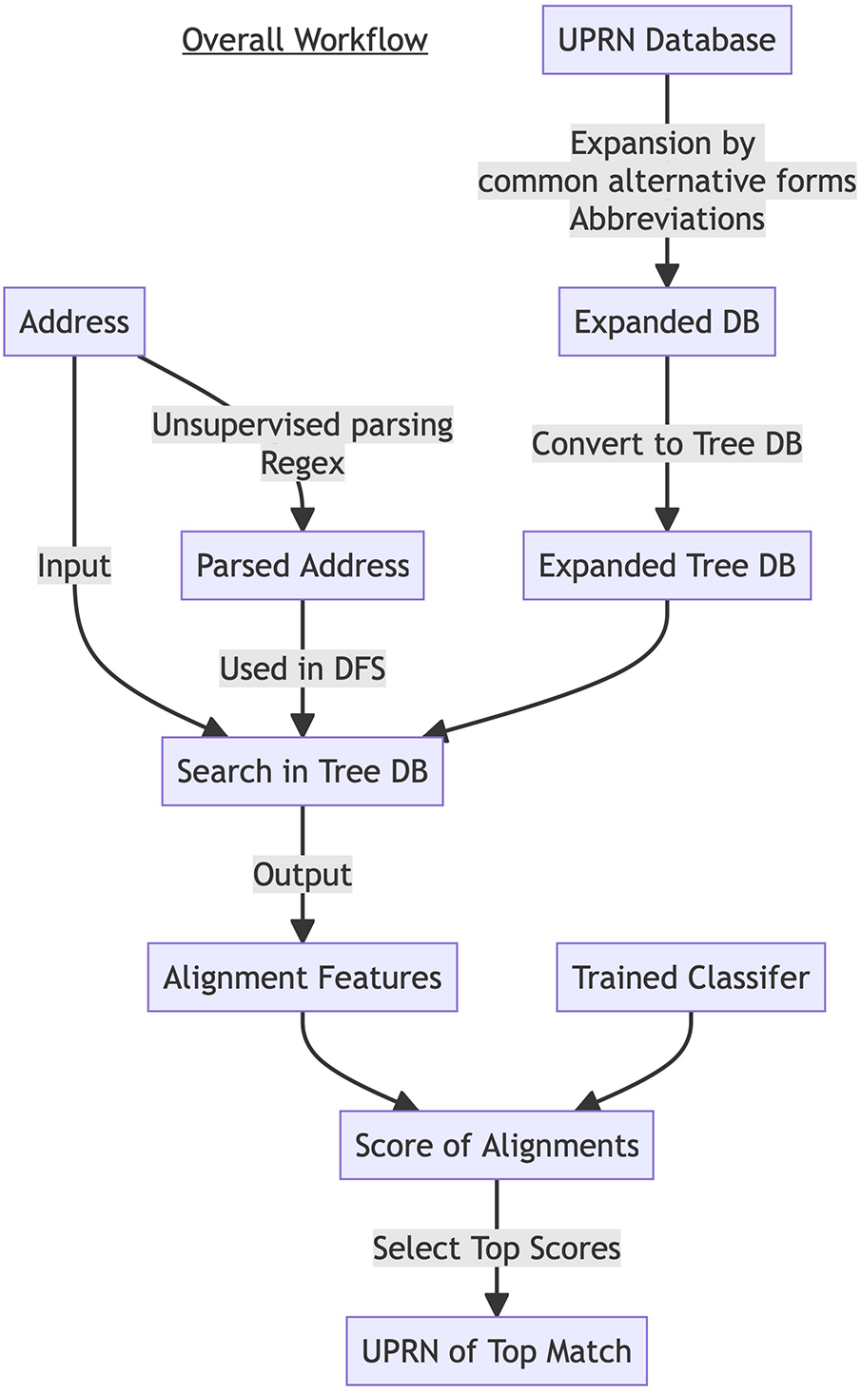
The Workflow of *FLAP*. UPRN DB is expanded by common alternative forms and abbreviations and converted to Tree DB. Each input address string is parsed by regular expression to extract postcode, post town, and thoroughfare. The address input string and parsed information were used to search in the Tree DB. Alignment features were generated for leaf nodes, each of which corresponds to a UPRN record. The alignment features were scored by a trained classifier. Top n- matched UPRN records were selected based on the scores.

An input address is preprocessed and then used to narrow down to the local area using the Tree DB and the alignment algorithm. Features for the *Random Forest Classifier* are generated for all pairs of the input address and records of addresses in the local area in the UPRN DB. Probabilities of matching are computed using the features and the trained classifier. The UPRN DB record with the highest probability is predicted to be the matching record (Figure 2).

### 2.8. Cohort

Addresses were extracted from CHI records for people registered at GPs in NHS Fife or NHS Tayside on 01 April 2017. In total, our data set contains 771,588 addresses of people registered with general practitioner practices in NHS Fife (363,091) and NHS Tayside (408,497).

### 2.9. Sampling training and test data set

The testing samples were selected from the addresses with stratified sampling by postcode. In each postcode, 12% of addresses were sampled (rounded up to the next integer), leading to the inclusion of 3,876 addresses. The training samples were selected with stratified sampling by postcode supplemented with oversampling of addresses that are likely to be ones of care homes. The reason is that care home addresses are often recorded more variably and, hence, provide enriched situations for model training. In total, 4,228 samples were included in the training data set. The test data set was not used in the development of *FLAP*.

### 2.10. Manual annotation of addresses in the cohort

Two annotators manually annotated each address in the training and test sets. The annotation involved the following steps: (1) If the address can be matched to a UPRN record using the best human effort, the UPRN number is allocated. The manual allocation allowed the use of external information (such as using the Royal Mail postcode finder and searching the address of a hotel name online). (2) If not, the address is labelled *not possible* and one of the following reasons is given: (a) *Not in database*: if the input address is in Royal Mail postcode finder but not in the current version of the UPRN database. (b) *Too broad*: if the input address corresponds to multiple records in the UPRN database. (c) *Low quality*: if the input address cannot be found in the Royal Mail postcode finder or is ambiguous.

Annotation was done independently by a general practitioner with experience in using addresses recorded in the input format in clinical practice (CM) and by a data scientist (HZ) with discrepancies resolved by discussions. The inter-annotator agreement (Cohen’s Kappa κ) was 0.980 before resolving the conflicts.

### 2.11. Performance assessment

For performance assessment, matching results were compared against the manual annotation.

We calculated the following performance metrics to assess the performance of *FLAP*:

• Raw accuracy: c/n
• Adjusted accuracy: c/p

where c is the number of addresses that are correct matches, n is the number of address samples, and p is the number of addresses that can be matched in manual annotation (see Section 2.10).

As described in Section 2.6, *FLAP* also outputs the probability of match as a score. We use the score to illustrate the usability aspect of *FLAP*. AccuracyinTop5 was defined as any correct mapping in the five candidates with the highest scores. Given the score s and the threshold of the score t, a cross-tabulation is created for the confidence score and matching correctness:

**Table:** 

	s>=t	s<t
Correct	tp	fn
Not Correct	fp	tn

The following metrics are calculated:

• *Precision* (or *Positive Predictive Value*): tp/(tp+fp)
• *Recall* (or *Sensitivity*): tp/(tp+fn)
• *F1*: harmonic mean of *Recall* and *Precision*

**Figure 3:**
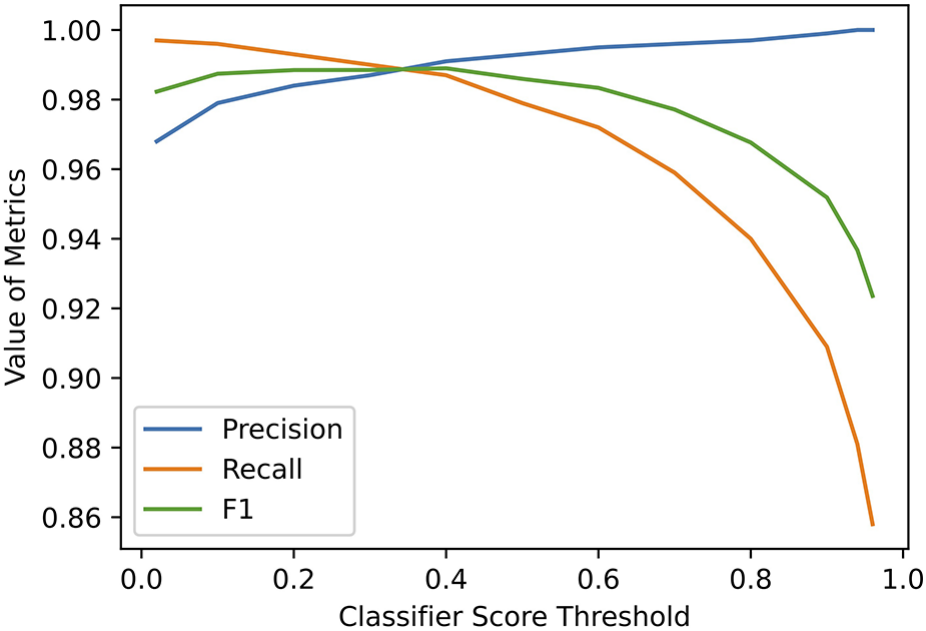
Performance of *FLAP* at different thresholds of classifier score.

A lift curve was generated to demonstrate the usability of *FLAP* at different confidence score thresholds (Figure 3).

## 3. Results

### 3.1. Performance of *FLAP*

The test samples contain 3,876 input addresses, of which 162 (4.18%) could not be matched to the UPRN database by human annotation. Among those not possible to match, 92 (56.8%) were not in the database, 25 (15.4%) were too broad, and 45 (27.8%) were of low quality (Table 2). *FLAP* achieved a raw accuracy of 0.950 (3,683/3,876) and adjusted accuracy of 0.992 (3,683/3,714) (Table 3). *FLAP* is able to handle common input variations like abbreviations, wrong postcodes, range-like addresses, and alternative forms. The number of successfully handled cases is summarised in Table 4. Among incorrectly matched, the most frequent reasons for wrong matching were: addresses requiring local knowledge for matching (7/31) (e.g., addresses with names of university halls and wrong postcodes with room numbers which can be mistaken as *BUILDING NUMBER*); partially incorrect information which happens to align with fields of other UPRN records (6/31); and incorrect information in both postcode and one of post town or thoroughfare (4/31).

**Table 2:** The baseline descriptive of input addresses in the test set.

Situation	Number (fraction in the test set)
Whole set	3,876 (1.000)
Possible to match	3,714 (0.958)
Of ones that are not possible	
Not in database	92 (0.024)
Too broad	25 (0.006)
Low quality	45 (0.012)

**Table 3:** Performance metrics of *FLAP*.

Metrics	Score
Raw accuracy	0.950
Adjusted accuracy	0.992
Adjusted accuracy in Top 5	0.994
Precision^a^	0.993
Recall^a^	0.983
F1^a^	0.988

^a^ Metrics shown here are calculated at the default threshold of t=0.5.

**Table 4:** Statistics where situations of input variations are handled.

Situation	Number (fraction)
Abbreviations	1,166 (0.313)
Wrong postcode	36 (0.097)
Range-like	8 (0.022)
Alternative form	6 (0.016)

### 3.2. Usability of *FLAP*

In addition to accuracy, it is important that *FLAP* outputs higher scores for correct matches. Precision, recall, and the F1 were calculated for a range of thresholds (Figure 3). At the default threshold of t=0.5, *FLAP* achieved a precision of 0.993, a recall of 0.983, and an F1 of 0.988 (Table 4). A customised threshold can be set to favour precision or recall, depending on the need of downstream applications.

Using the confidence scores for matching, we could select the top five candidates from UPRN DB for matching to an address. The rate of finding any correct matching in the top five candidates (accuracy in top 5) is 0.994 (in addresses that are possible to match) up from 0.992 for choosing the candidate with the highest score.

We performed a runtime analysis of *FLAP* to demonstrate the usability in scenarios involving large amount of data. On average, *FLAP* can process 6.94 addresses per second on an Intel i7-12700k CPU (or 144 ms per address) (Table 5). Parsing and matching of the address take 34.1% and 65.9% of the runtime, respectively. For the 771,588 GP-registered in Tayside and Fife, the total runtime was under 31 h.

**Table 5:** Comparison of existing non-proprietary methods.

Tool	*FLAP*	ASSIGN	CURL
Type	Probabilistic	Deterministic	Deterministic
Match method	Machine learning	Rules	Rules
Reported match rate in test data set	0.950/0.992^a^	0.986^b^	0.890^b^
Average runtime per address	144 ms	1.58 ms^c^	Not reported
Manual annotation in test data set	+	−	−
Adaptability to new data	+	−	−
Allow continuous improvement	+	−	−
Allow local knowledge	+	−	−
Output matching quality	+	+	+?
Independent components	+	?	?
Compare to ground truth in training data set	+	+	−
Potential of semantic matching	+	−	−

^a^ These match rates are raw accuracy and adjusted accuracy,respectively.

^b^ These match rates did not consider accuracy of match or if a match is possible.

^c^ The hardware specification was not reported.

## 4. Discussion

In this article, we described the *FLAP* framework, a generalisable probabilistic record linkage tool based on machine learning for linking UK addresses to UPRN DB entries. We have achieved state-of-the-art performance in real-world data with more comprehensive evaluation metrics and improved usability aspects (Table 5).

*FLAP* employed machine learning algorithms as the classifier for record-matching decisions. The use of machine learning improves the address linking task in three ways: (1) The probabilistic output allows quality control of linkage by adjusting the threshold of matching probability and output of top n candidates of matching. Although, including top five candidates only led to a marginal increase of adjusted accuracy (0.002) in our data set, this feature is potentially useful when *FLAP* is applied to unseen data, where we expect a drop of accuracy for top one matching. In a real-world scenario, being able to query the top n match for an address saves one from the time-consuming job of matching to the entire database. (2) When applying *FLAP* to unseen data, the machine learning classifier has the flexibility to be further trained using new data, which is difficult for rule-based methods. (3) The machine learning classifier can be continuously improved during use with a stream of newly labelled data.

Among the three existing non-proprietary tools, our study is the only one which measures accuracy against a test data set, instead of reporting only the percentage of matched addresses (Table 5). We believe that our approach provides more transparent performance evaluation of the linking algorithm, although external validation using data from other regions in the UK is still required.

We demonstrated our database augmentation strategy dealt with common input variations like abbreviations, range-like addresses, and alternative forms. The database augmentation process is compatible with the manual curation of non-standard addresses, which normally requires “local knowledge.” These addresses may not be possible to be matched otherwise since a non-standard address may share no similarity to records in the UPRN DB textually or semantically (e.g., Name of a local hotel).

The current implementation of *FLAP* uses blocking by exact filtering of postcode or post town/thoroughfare pair. The blocking strategy is time-efficient but does not work if neither a correct postcode nor a correct post town/thoroughfare combination is present in the address input. Although such cases are empirically rare, this limitation could be addressed in the future by using blocking techniques that are independent of parsing such as locality sensitive hashing (LSH). Records for pair-wise can be narrowed down by approximate nearest neighbour searching (ANNS) (14).

To apply *FLAP* on new data, manual labelling of data is still needed to ensure optimised performance. However, the data labelling can be accelerated using the existing model. In our experience, a trained annotator could generate an effective labelled dataset for a new task within one day.

The semantics of words were not yet considered in matching. Theoretically, the semantics embedding of words will improve linking when two words with similar meanings but differences in string similarity metrics are often used interchangeably. This issue is more important in linking of care home addresses, where addresses often include related words like “home” and “house.” *FLAP* is set up in a way that semantic similarity matching can be integrated and tested. For example, semantic similarity can be calculated from pre-trained word embedding models [e.g., *GloVe* (15) or *Word2Vec* (16)] and used in token-based alignment in combination with string similarly metrics. This aspect will be tested in future work (Table 5).

The order of UPRN fields being aligned to an input address is set empirically at the moment. UPRN fields that are empirically more structured in input address (like postcodes) were aligned in priority. However, the alignment of fields is not always optimised, since conflicts of alignments between fields are not resolved optimally (e.g., names of post towns could be aligned to street names). For this reason, alignment optimisation considering all possible alignments of UPRN fields to an input address might improve the performance of alignment. A possible implementation of such is linear assignment algorithm (17), in which we can consider each UPRN field to be a worker and the score (which will have to be a distance-like score to fit in the cost minimisation framework) of each possible alignment to be the cost.

The runtime requirement of *FLAP* is significantly higher than ASSIGN (9) due to more computation steps involved, although hardware specification was not reported for ASSIGN. *FLAP* can process approximately 600,000 address records per day on a mid-end CPU and can be further accelerated by adding more computing resources. The runtime can be further improved by employing deterministic rules for addresses that are easy to match.

Finally, *FLAP* does not currently support one-to-many linking of an input address to UPRN records. This issue applies to input addresses that are too broad so that the input address covers multiple records in the UPRN database. Since different frameworks and performance assessment metrics are needed for one-to-many record linkage, this problem is out of the scope of this paper and will be addressed in future work.

In conclusion, we have developed a framework, *FLAP*, for linking free-text UK addresses to UPRN DB with good real-world performance and usability.

## Data Availability

Licence for UPRN © Crown copyright and/or database rights 2023 OS (Research Licence). This article used data created and maintained by Scottish Local Government.

## Data availability statement

The datasets presented in this article are not readily available because they can only be accessed by submitting an application to the information governance body: the Health Informatics Centre of the University of Dundee. Requests to access the datasets should be directed to b.alex@ed.ac.uk.
