# Supplementary material for: FLAP: a framework for linking free-text addresses to the Ordnance Survey Unique Property Reference Number database
Source: Front Digit Health. 2023 Nov 28;5:1186208. doi: 10.3389/fdgth.2023.1186208 (PMC10715280; doi:10.3389/fdgth.2023.1186208)
Supplement: Supplementary file 1 [file Datasheet1.pdf]

Supplementary Material

1 SUPPLEMENTARY TABLES AND FIGURES

1.1 Figures

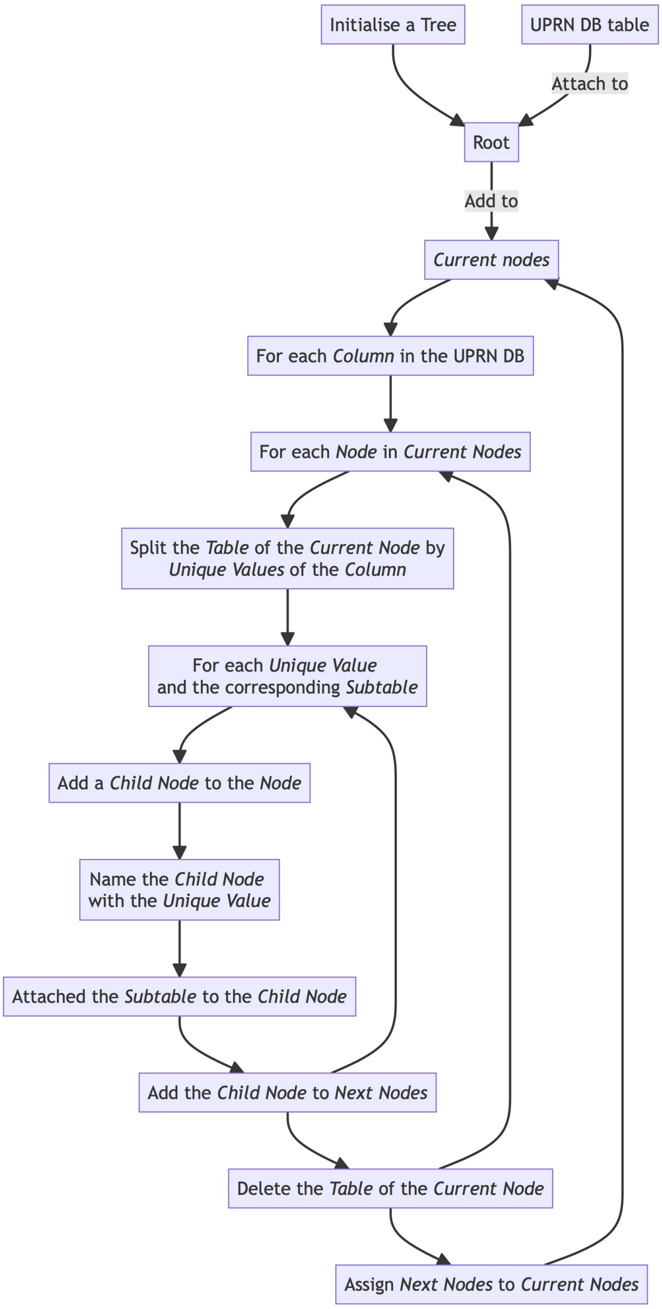

Figure S1. Process of UPRN DB to Tree DB conversion

1.2 Tables

Type	Regex	In fields	Out fields	Example
Range-like BUILDING NAME	`^([A-Z ]+)?(\d+)([A-Z])?-(\d+)([A-Z])?([A-Z ]+)?$`	BUILDING NAME	BUILDING NUMBER	“1-3” to [“1”, “3”]
Compound BUILDING NAME	`(\d+)([A-Z]|/\d+)`	BUILDING NAME	SUB BUILDING NAME and BUILDING NUMBER	“18A” to “FLAT A 18”
Common SUB BUILDING NAME	`(FLAT|UNIT) (\d+[A-Z]?|[A-Z])`	SUB BUILDING NAME and BUILDING NUMBER	BUILDING NAME	“FLAT A 18” to “18A”
Synonyms	(*SYNONYM*)	Any	Any	“HIGH STREET” to “HIGH ST”

Table S1. Regular expression rules for UPRN DB augmentation

Original	Synonyms
ROAD	RD
STREET	ST
PLACE	PL
CRESCENT	CRES
DRIVE	DR
AVENUE	AVE
TERRACE	TER
GARDENS	GDNS
GARDEN	GDN
COURT	CT
PARK	PK
ST.	ST
GROVE	GR, GRO
SOUTH	S
WEST	W
NORTH	N
WALK	WLK
EAST	E
SQUARE	SQ
CLOSE	CL
CHURCH	CH
SCHOOL	SCH
FLAT	FLT, FL
APPARTMENT	APT
APPARTMENTS	APTS
FARM	FM
HOUSE	HSE
ACCOMMODATION	ACC
LIEUTENANT	LT
LODGE	LDG
COTTAGE	COTT

Table S2. List of synonyms used for database augmentation
